# Sequential Use of Second-Generation Tyrosine Kinase Inhibitor Treatment and Intensive Chemotherapy Induced Long-Term Complete Molecular Response in Imatinib-Resistant CML Patient Presenting as a Myeloid Blast Crisis

**DOI:** 10.1155/2017/3209305

**Published:** 2017-12-17

**Authors:** Masaaki Tsuji, Tatsuki Uchiyama, Chisaki Mizumoto, Tomoharu Takeoka, Kenjiro Tomo, Tatsuharu Ohno

**Affiliations:** Department of Hematology and Immunology, Otsu Red Cross Hospital, 1-1-35, Nagara, Otsu-shi, Shiga, Japan

## Abstract

Myeloid blast crisis of chronic myeloid leukemia (CML-MBC) is rarely seen at presentation and has a poor prognosis. There is no standard therapy for CML-MBC. It is often difficult to distinguish CML-MBC from acute myeloid leukemia expressing the Philadelphia chromosome (Ph+ AML). We present a case in which CML-MBC was seen at the initial presentation in a 75-year-old male. He was treated with conventional AML-directed chemotherapy followed by imatinib mesylate monotherapy, which failed to induce response. However, he achieved long-term complete molecular response after combination therapy involving dasatinib, a second-generation tyrosine kinase inhibitor, and conventional chemotherapy.

## 1. Introduction

The prognosis of patients with myeloid blast crisis of chronic myeloid leukemia (CML-MBC) is extremely poor, with a median survival time of only a few months [1]. Imatinib monotherapy for CML-MBC produces comparable results to conventional chemotherapy, but the associated survival period remains short [2, 3]. A recent study showed that the use of imatinib has improved the outcomes of blast crisis of CML (CML-BC) to some extent, but the median survival time of patients with the condition is still short (6.5–10 months) [4]. The use of second-generation tyrosine kinase inhibitors (2nd TKIs) such as dasatinib and nilotinib to treat CML-MBC has also resulted in improved outcomes [5–9]. Furthermore, combination treatment with conventional chemotherapy and TKIs is currently being investigated and is expected to result in better outcomes [11–13].

When the proliferation of Philadelphia chromosome-positive (Ph+) blasts is detected at presentation, it can be difficult to distinguish between CML-MBC and Ph+ acute myeloid leukemia (AML). Although several studies have detected clinical, pathological, and cytogenetic differences between CML-MBC and Ph+ AML, it remains uncertain whether these differences represent true entity-defining characteristics or merely reflect a more rapid clinical presentation of the same disease [14, 15].

Here, we report a case in which a patient with conventional chemotherapy- and imatinib-resistant CML-MBC achieved a long-term complete molecular response (CMR) after sequential treatment with a 2nd TKI and conventional chemotherapy.

## 2. Case Report

**Figure 1:**
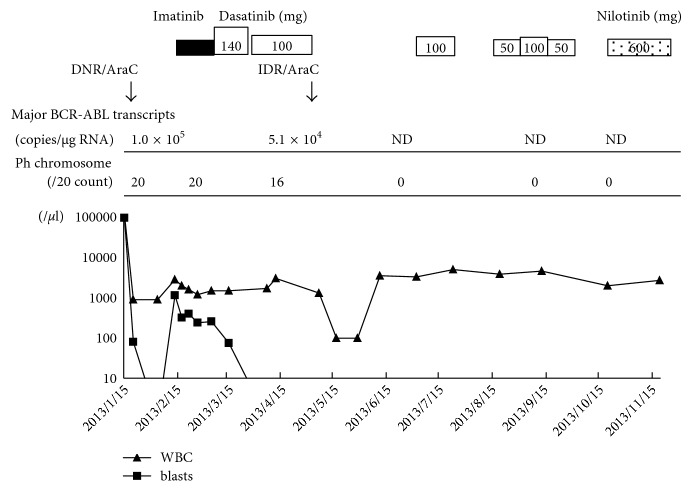
Clinical course. The kinetics of the hematological, cytogenetic, and molecular responses to tyrosine kinase inhibitors and conventional chemotherapy seen during the patient's clinical course are shown. Ara-C: cytosine arabinoside, DNR: daunorubicin, IDR: idarubicin, and ND: not detectable.

A 75-year-old male was admitted to our hospital due to a month-long history of malaise. He had not previously suffered from hematological disorders. However, he had diabetes and was an active smoker. On physical examination, he exhibited mild splenomegaly but not lymphadenopathy. His initial white blood cell count was as follows: 113,500/*µ*L with 83% blasts, 1% promyelocytes, 2% myelocytes, 2% metamyelocytes, 0% stabs, 2% segmented neutrophils, 0% basophils, 0% eosinophils, 32% monocytes, and 7% lymphocytes. His hemoglobin level and platelet count were 8.8 g/dL and 2.1 × 10^4^/*µ*L, respectively. His serum lactate dehydrogenase level was 2,162 U/L. Bone marrow aspiration disclosed a hypercellular marrow with a blast cell frequency of 83.8% (Table 1). The blast cells were positive for myeloperoxidase (MPO). No dysplastic features were observed in the patient's erythroid or megakaryocyte cells. Flow cytometric analysis of the bone marrow blasts produced the following results: CD13+: 94.2%, CD33+: 25.0%, CD117+: 71.9%, MPO+: 4.6%, and human leukocyte antigen- (HLA-) DR+: 90.0%. A cytogenetic study of the same material revealed the following karyotype in 100% (20/20) of the cells: 46,XY,*t*(9;22)(*q*34;*q*11.2). Fluorescence in situ hybridization (FISH) using the bcr–abl probe produced positive results for both the interphase peripheral polymorphonuclear cells (74.4%) and mononuclear cells (94.1%). The patient's bone marrow cells contained 1.0 × 10^5^ copies/*μ*g RNA of the major BCR-ABL mRNA molecule, detected by real-time quantitative reverse transcription polymerase chain reaction (RQ-PCR) (Table 1). The results of the FISH analysis and splenomegaly were more suggestive of CML-MBC than de novo Ph+ AML. The patient initially received intensive chemotherapy with 50 mg/m^2^ daunorubicin on days 1–3 and 100 mg/m^2^ cytosine arabinoside (Ara-C) on days 1–7. He did not respond to induction chemotherapy. He subsequently received 600 mg/day imatinib alone. Three weeks after the initiation of imatinib therapy, the number of blast cells in the patient's peripheral blood was increased, indicating that the imatinib treatment had not resulted in hematological remission. At that time, imatinib and dasatinib were the only TKIs approved in Japan for use in CML-BC. Thus, dasatinib treatment (140 mg daily) was started. About 20 days later, the patient developed grade 3 pleural effusion as a side effect of dasatinib. After the discontinuation of dasatinib and diuretic treatment, he recovered. Dasatinib was restarted at a dose of 100 mg/day, and the patient achieved a complete hematological response (CHR) within 34 days. After 42 days, bone marrow aspiration was performed. Cytogenetic analysis of the bone marrow revealed the Ph chromosome in 80% (16/20) of the cells, and BCR-ABL was detectable at the molecular level (5.1 × 10^4^ copies/*μ*g RNA). The patient was treated with reinduction chemotherapy (12 mg/m^2^ idarubicin on days 1–3 and 100 mg/m^2^ Ara-C on days 1–5) after the cessation of dasatinib treatment. After the bone marrow had recovered, bone marrow aspiration confirmed that hematological, immunophenotypic, and cytogenetic remission had been achieved, and no BCR-ABL transcripts were detected by RQ-PCR-based analysis of the bone marrow aspirate. Furthermore, FISH analysis using the bcr–abl probe produced negative results for both the interphase peripheral polymorphonuclear cells and mononuclear cells. Consolidation chemotherapy was not administered because the patient developed a severe pulmonary infection. As no matched sibling donor was available, and the patient's comorbidities were correlated with a hematopoietic cell transplantation-specific comorbidity index of 3, he was not eligible for an allogeneic hematopoietic stem cell transplantation (allo-SCT). Therefore, dasatinib monotherapy was started at a dosage of 100 mg/day. About one month later, he developed bilateral pleural effusion as a side effect of the dasatinib therapy. After the discontinuation of the dasatinib and diuretic treatment, he recovered. Dasatinib treatment was reinitiated at a dose of 50 mg/day, but pleural effusion developed again. Moreover, grade 4 thrombocytopenia and grade 4 neutropenia also arose. Thus, dasatinib was replaced with nilotinib at a reduced dosage of 600 mg/day (Figure 1). At 3.5 years after the initiation of nilotinib treatment, no BCR-ABL transcripts were detected by RQ-PCR-based analysis of the patient's bone marrow aspirate. We also confirmed that there were no detectable BCR-ABL transcripts in the patient's peripheral blood (a molecular response of > MR4.5 according to the International Scale). He is still in CMR and is being treated with nilotinib at a daily dose of 600 mg.

## 3. Discussion

Myeloid blast crisis is the major remaining problem in the management of CML. In a multicenter study of imatinib monotherapy for CML-MBC, only 15.3% of the patients achieved CHR, and the median survival time was 6.9 months [2]. In another study with a 6-year follow-up period, 72 patients with CML-MBC were treated with imatinib alone at a dosage of 600 mg/day. CHR were achieved in 24% of cases, complete cytogenetic responses were achieved in 5% of cases, and the median survival time was 7 months [3]. Several studies have assessed the use of a combination of imatinib and conventional chemotherapy to treat CML-MBC. In a phase I/II trial involving 16 CML-MBC patients, 600 mg imatinib daily was combined with mitoxantrone and etoposide. The median overall survival time was 6.4 months [10]. Another study described the use of a combination of 600 mg imatinib and low-dose Ara-C and idarubicin in 19 CML-MBC patients. The median survival time was only 5 months [11]. Deau et al. reported the outcomes of 36 patients with CML-MBC that were treated with daunorubicin and Ara-C in combination with imatinib. The median overall survival time was 16 months, and the results were significantly improved by escalating the daily dose of daunorubicin [12]. These results suggest that the use of a more intensive induction regimen in combination with imatinib might improve the outcomes of CML-MBC.

The use of 2nd TKIs could overcome the refractoriness of CML-MBC to conventional chemotherapy, including imatinib therapy. Compared with imatinib therapy, several studies of CML-BC patients that were treated with dasatinib therapy showed hematological remission rates of 33% to 61%, major cytogenetic remission rates of 35% to 56%, and median survival times of 8 to 11 months [5–7]. In a CML-MBC cohort study, Saglio et al. found that the median overall survival time was 7.7–7.9 months [7]. Two studies of CML-BC patients that were treated with nilotinib therapy have been published [8, 9]. Giles et al. reported that CML-MBC patients exhibited a hematological remission rate of 60%, a major cytogenetic remission rate of 38%, and a median survival time of 10.1 months [9]. Thus, the combination of a 2nd TKI with conventional chemotherapy as a treatment for CML-MBC is of interest. A phase II study of Ph+ acute lymphoblastic leukemia and the lymphoid blast phase of CML involving patients that were treated with a combination of the hyper-CVAD (cyclophosphamide, vincristine, doxorubicin, and dexamethasone) regimen and dasatinib detected a complete cytogenetic remission rate of 84% and a complete molecular response rate of 42%. Although some grade 3 and 4 toxicities, including episodes of bleeding (34%), pleural effusion (15%), and pericardial effusion (6%), arose, the treatment regimen was well tolerated [16]. Milojkovic et al. reported that dasatinib can be safely combined with conventional chemotherapy, such as the FLAG-IDA (fludarabine, Ara-C, granulocyte colony-stimulating factor, and idarubicin) regimen, and this combination seems to induce deep remission in patients with CML-BC [13].

The incidence of Ph+ AML is reported to be 0.35–0.9% of all cases of AML. Whether Ph+ AML is distinct from CML-MBC was disputed until recently. Various clinical characteristics have been suggested to be helpful for differentiating Ph+ AML from CML-MBC, including the absence of previous myeloproliferative disorders, the absence of evidence of chronic- or accelerated-phase CML, and a lack of clinical and laboratory features of CML, such as splenomegaly, basophilia, and a higher bone marrow myeloid/erythroid ratio. In addition, compared with CML-MBC, Ph+ AML is associated with very marked leukocytosis. In a FISH analysis of polymorphonuclear cells, the p210 BCR-ABL protein was found to be more common than the p190 BCR-ABL protein in Ph+ AML. The presence of additional cytogenetic aberrations, such as an extracopy of the Ph chromosome, trisomies 8 and 19, and isochromosome 17*q*, is more common in CML-MBC than in Ph+ AML. The coexistence of normal metaphases in addition to Ph+ metaphases at presentation is more characteristic of Ph+ AML than of CML-MBC. Moreover, the persistence of *t*(9;22) after induction therapy is more common in CML-MBC than in Ph+ AML [14]. A recent study showed that nucleophosmin 1 (NPM1) mutations are seen exclusively in Ph+ AML, whereas ABL1 mutations are exclusive to CML-MBC, suggesting that Ph+ AML is distinct from CML-MBC [15]. In our case, at the time that a dasatinib-induced CHR was achieved, cytogenetic analysis of the patient's bone marrow revealed the Ph chromosome in 80% (16/20) of the cells, and FISH analysis using the bcr–abl probe produced positive results for both the interphase peripheral polymorphonuclear cells and mononuclear cells. The patient's clinical course also supported a diagnosis of CML-MBC.

Our patient has been in CMR for more than three years. He has continued to take nilotinib after dasatinib, and intensive chemotherapy produced a CMR. Although an allo-SCT remains the only curative option for patients with CML-MBC, long-term remission may be expected by administering a combination of 2nd TKIs and intensive chemotherapy even without allo-SCT, given that the patient cannot tolerate the procedure or no suitable donor was available.

## Figures and Tables

**Table 1:** Laboratory data on admission.

CBC		Chemistry		Bone marrow	
WBC	113,500/*μ*l	LDH	2,162 U/l	NCC	1,30,000/*μ*l
Blasts	83.0%	AST	74 U/l	Megakaryocytes	0.0/*μ*l
Promyelocytes	1.0%	ALT	444 U/l	M/E	11.40
Myelocytes	2.0%	ALP	250 U/l	Blasts	83.8%
Metamyelocytes	2.0%	T-Bil	0.59 mg/dl	Promyelocytes	1.0%
Stabs	0.0%	T-Pro	6.0 g/dl	Myelocytes	1.0%
Segmented	2.0%	Na	139 mEq/l	Metamyelocytes	0.6%
Eosinophils	0.0%	K	4.3 mEq/l	Slabs	1.4%
Basophils	0.0%	Cl	107 mEq/l	Segmented	1.0%
Monocytes	32.0%	BUN	17.9 mg/dl	Eosinophils	0.0%
Lymphocytes	7.0%	Creatinine	1.18 mg/dl	Basophils	0.0%
Reticulocytes	5.5%	CRP	1.2 mg/dl	Monocytes	1.2%
RBC	289 × 10^4^/*μ*l	—	—	Lymphocytes	2.0%
Hemoglobin	8.8 g/dl	—	—	G-banding	46,XY,*t*(9;22)(*q*34;*q*11.2)
PLT	2.1 × 10^4^/*μ*l	—	—	Major bcr/abl mRNA	1.0 × 10^5^ copies/μg RNA

CBC: complete blood cell count, WBC: white blood cells, RBC: red blood cells, PLT: platelets, LDH: lactate dehydrogenase, AST: alanine aminotransferase, ALT: aspartate aminotransferase, ALP: alkaline phosphatase, T-Bil: total bilirubin, T-Pro: total protein, BUN: blood urea nitrogen, CRP: C-reactive protein, NCC: nuclear cell count, and M/E: myeloid to erythroid ratio.
